# A Case of Post-malarial Postural Orthostatic Tachycardia Syndrome in a Young Woman of South Asian Origin

**DOI:** 10.7759/cureus.71606

**Published:** 2024-10-16

**Authors:** Mousumi Barua

**Affiliations:** 1 Internal Medicine, School of Public Health and Health Professions, University at Buffalo, Buffalo, USA

**Keywords:** beta-blockers, dyspnea, malignant malaria, orthostatic intolerance, postural orthostatic tachycardia syndrome (pots), syncope, tachycardia, thermoregulation, tilt table test, vasoactive agents

## Abstract

Postural orthostatic tachycardia syndrome (POTS) often manifests following an acute infection. This case study reports a post-malarial onset of POTS in a young female medical student who presented with tachycardia, dizziness, presyncope, several episodes of syncope, and dysautonomic symptoms. The diagnosis of POTS was established only four years later by a tilt table test. The patient made a remarkable functional recovery from POTS, as she responded well to pharmacotherapy, primarily beta-blockers, along with lifestyle modifications. Although mild dysautonomic symptoms such as lack of thermoregulation and decreased perspiration have persisted throughout her life, the patient has managed to cope with these symptoms and function well in her daily life. To our knowledge, this is probably the first reported case of post-malarial POTS, and we recommend that the non-viral etiology of POTS, particularly the post-malarial cases, be studied in detail to understand the pathophysiology and management of this disorder.

## Introduction

Postural orthostatic tachycardia syndrome (POTS) is a dysautonomic syndrome [1] characterized primarily by impairments in autonomic control of cardiovascular, gastrointestinal, and thermoregulatory functions [2, 3], resulting in symptoms of orthostatic intolerance, including anxiety/palpitation, dizziness/vertigo, presyncope/syncope, hyperventilation/dyspnea, tremulousness, sweating, headache, fatigue, sleep disorders, and weakness [4]. While POTS patients can present with a myriad of symptoms, the most common ones include lightheadedness (99%), tachycardia (97%), presyncope (94%), headache (94%), and difficulty concentrating (94%) [5]. Biometrically, POTS is diagnosed when there is a sustained heart rate increase of ≥30 beats per minute (bpm) or an increase in heart rate to ≥120 bpm within the first 10 minutes of orthostatic intolerance without significant orthostatic hypotension [6]. Postural orthostatic tachycardia syndrome can be chronic and debilitating [3], and symptoms can impact patients’ ability to perform daily activities, such as bathing, housework, and feeding [4]. The prevalence of POTS is approximately between 0.2% and 1% of the population, most commonly diagnosed in young and middle-aged women as well as in individuals with chronic fatigue syndrome or autoimmune disorders [3]. While POTS can occur spontaneously due to an unknown or genetic etiology [7], it can also develop in response to viral infections, including COVID-19 and other medical conditions, such as Ehlers-Danlos syndrome, Lyme disease, or autoimmune disorders [8].

Based on the consensus statements [9, 10], POTS is formally defined as a clinical syndrome lasting at least six months that is characterized by (i) an increase in heart rate ≥30 bpm within five to 10 minutes of quiet standing or upright tilt (or ≥40 bpm in individuals 12 to 19 years of age), (ii) the absence of orthostatic hypotension (>20 mmHg drop in systolic blood pressure), and (iii) frequent symptoms that occur with standing such as lightheadedness, palpitations, tremulousness, generalized weakness, blurred vision, exercise intolerance, and fatigue [11]. While the diagnostic label and criteria of POTS were formally established in the 1990s [11], many disorders with similar symptom clusters have been described in published reports for over 150 years by various other diagnostic terms, including DaCosta’s syndrome, irritable heart, soldier’s heart, effort syndrome, neurocirculatory asthenia, and mitral valve prolapse syndrome [12]. Several subtypes of POTS, such as neuropathic POTS, autoimmunity, hyperadrenergic POTS, mast cell activation disorder, and volume dysregulation, have been proposed [11]. Although POTS has been identified as a heterogeneous and multifactorial disorder [13], the pathophysiological mechanisms underlying the disorder are not fully understood [11]. Despite the heterogeneity of symptoms and manifestations of POTS, a ubiquitous symptom of POTS is cardiovascular deconditioning [14], characterized by cardiac atrophy and hypovolemia, which is a major cause of functional disability [11].

While there is growing interest in POTS research [3], especially after the COVID-19 pandemic [15], there are significant challenges in studying POTS for many reasons [3], including its low prevalence rate, unsuccessful clinical trials, difficulty diagnosing due to variations of similar syndromes, and lack of consistency in outcome measures. Furthermore, while there is a surge in interest in studying post-COVID manifestations of POTS [15], studies related to other etiological factors, for example, post-malarial POTS, have not been reported, although malaria-induced neural damage caused by Plasmodium falciparum has been previously reported [16]. It is highly likely that such cases, which could have been otherwise diagnosed as POTS, have been underreported as there is a severe lack of studies. To our knowledge, this is the first report of the post-malarial onset of POTS. Recognizing POTS as sequelae induced by malarial infection is important in order to provide appropriate treatment, especially to those in poor tropical countries prone to frequent malaria outbreaks.

With this background, we present here a case report about a 30-year-long clinical history of a woman who initially developed POTS symptoms (i.e., tachycardia, syncope, and dysautonomia) following an acute episode of malignant malaria and her dramatic recovery from her major symptoms and near-normal functioning in many facets of her life.

## Case presentation

First hospital visit (at age 25)

**Figure 1:**
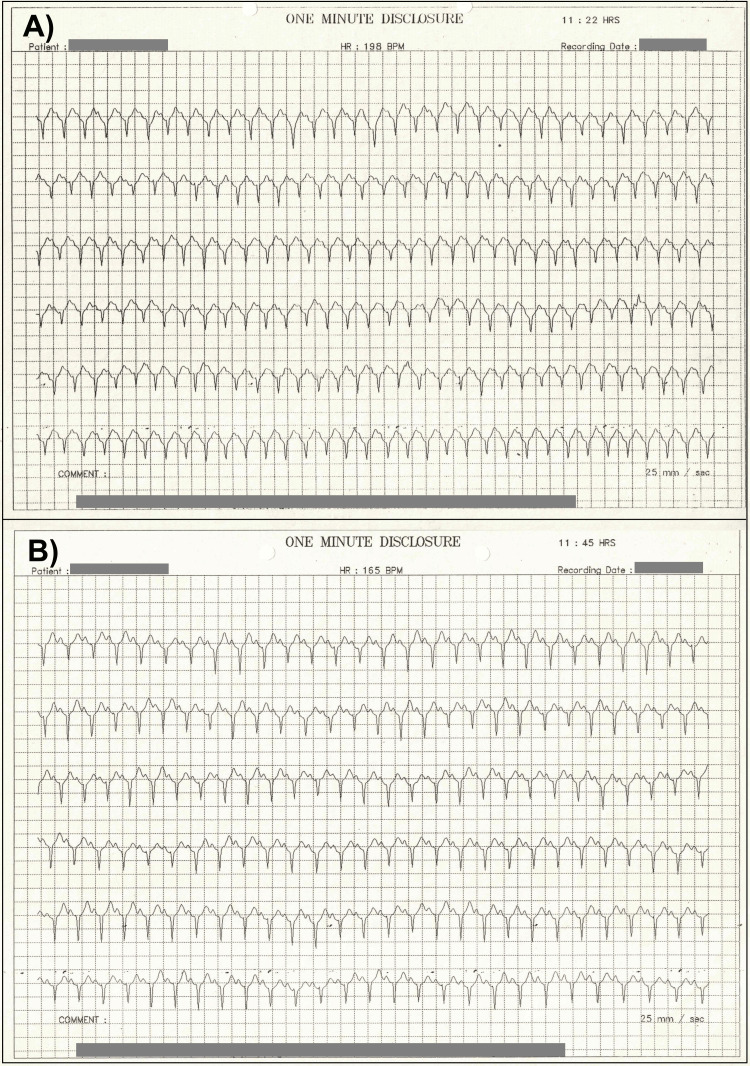
Two sample segments taken from the Holter ECG recording A) A segment at 11:22 hours showing 198 beats per minute (bpm); and B) A segment at 11:45 hours showing 165 bpm. Note that the patient’s identifiable information has been masked out.

A 25-year-old right-handed female medical student presented at a multispecialty hospital in southern India with a history of recurrent attacks of palpitations associated with giddiness and syncope. These symptoms started after her recovery from a fatal episode of malignant malaria caused by *Plasmodium falciparum* four years ago (at age 21) when she was a first-year medical student. She was treated with antimalarial therapy, primarily intravenous quinine and IV fluids. Her malarial illness lasted about three weeks, and she lost 14 kg of body weight (from 48 kg to 34 kg). After about four weeks of recovery from malaria, the patient started having recurrent attacks of palpitation, which at times led to episodes of presyncope and syncope characterized by loss of consciousness and fainting or passing out. Her medical education was interrupted because of these symptoms. At times, her heart rate went beyond 165 to 198 bpm, followed by a dizzy spell, and terminated by collapse. Syncopal attacks became more frequent over the past year after her first hospital visit (seven times overall). On clinical examination, she had a structurally normal heart. Her thyroid function was normal, and other blood investigations did not reveal any abnormalities. The 24-hour ECG from the Holter monitor report showed evidence of atrial tachycardia (Figure 1).

**Figure 2:**
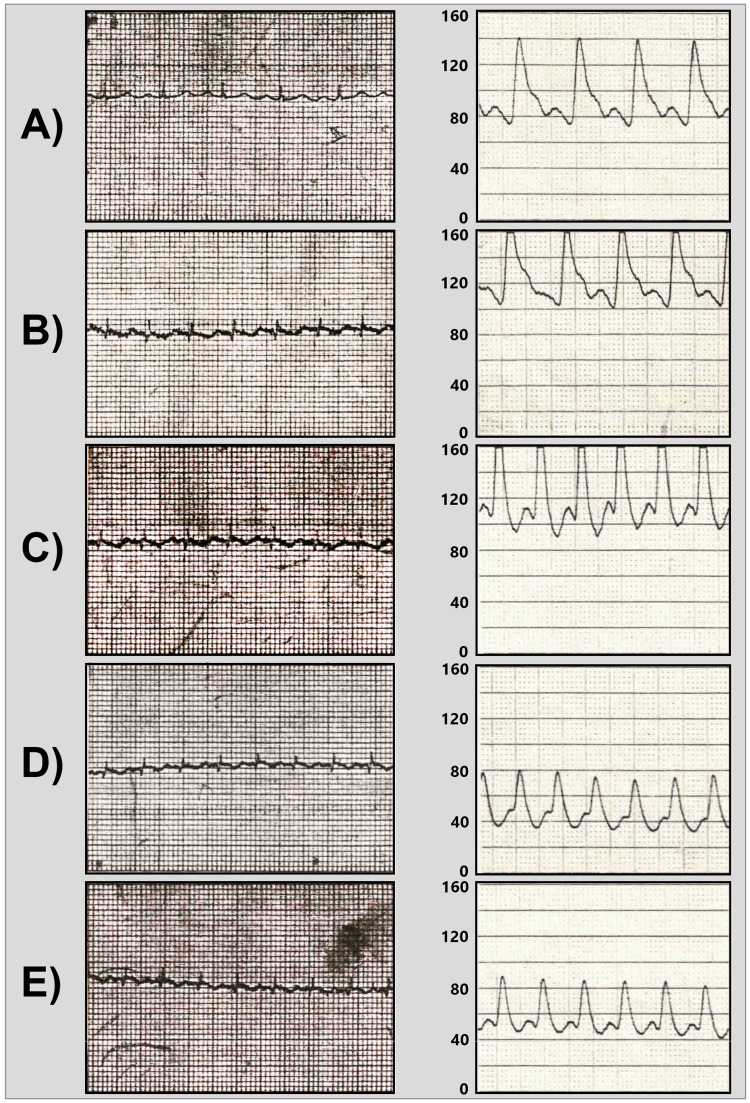
Recording of ECG rhythm (left panels) and intra-arterial blood pressure (right panels) during the tilt testing at various positions and stages A) 0° tilt position during rest; B) 90° tilt position during rest; C) 0° tilt position during isoprenaline challenge; D) 90° tilt position during isoprenaline challenge; and E) 0° tilt position during the post-isoprenaline stage. Note that the Y-axis of the blood pressure graphs on the right side ranges 0-160 mmHg, and each segment of the X-axis represents time in seconds.

Based on the Holter EEG findings, a provisional diagnosis of inappropriate atrial tachycardia was made. To further rule in/out the diagnosis of POTS, the patient was further subjected to tilt table testing, which entailed continuous monitoring of ECG rhythms and intra-arterial pressure under various conditions (Figure 2). Her heart rate at rest was around 100-120 beats per minute (bpm), and it increased progressively, while her blood pressure response was normal. During the intravenous isoprenaline challenge, the patient developed sustained atrial tachycardia, and her blood pressure dropped after about five minutes of continuous tachycardia. Soon after, the patient’s symptom cluster was reproduced as she felt a dizzy spell and nearly collapsed. Her blood pressure response became normal after resting for about 10 minutes, and her heart rate settled to a sinus rhythm at a rate of 100 bpm.

The findings of the tilt table test confirmed the diagnosis of POTS, which was considered a rare and novel clinical condition at that time as there were only a few reports on POTS. The patient was prescribed atenolol 25 mg twice a day (which is primarily used to treat angina and hypertension). The dosage could be increased to 50 mg twice a day if needed, along with salt therapy. If this regimen did not help her to a great extent, a small dose of fludrocortisone (a steroid that would raise her blood pressure to the normal range in the body) was prescribed, which could be supplemented with beta-blocker therapy as needed. Soon after, atenolol 25 mg twice a day (BID) was replaced with metoprolol 50 mg twice daily, as there was no significant improvement in controlling the heart rate. Since midodrine was not available in India at that time, the patient was also prescribed oral fludrocortisone (Florinef) to maintain her blood pressure. She was also prescribed diltiazem XR 30 mg once a day (QD) and Deriphyllin 150 mg for symptom control. With regular medication, the patient improved dramatically and without having major episodes of syncope, although mild symptoms of palpitation, dizziness, and hypersensitivity to extreme temperatures persisted. The patient was able to cope with these symptoms by implementing lifestyle changes as needed. She went on to complete her medical education and started working as a physician, as these symptoms did not disrupt her daily activities or occupational functioning.

Second hospital visit (at age 33)

Following her dramatic improvements in her symptoms thanks to her correct diagnosis and treatment, the patient married at the age of 31 and migrated to the United States. Within a year of her happy marriage, she had a healthy child despite undergoing a high-risk pregnancy and cesarean birth. However, around the age of 28 years, she suffered symptoms of severe gastroparesis, thrived mostly on a liquid diet, and lost approximately 15 pounds of body weight. Furthermore, she occasionally developed symptoms of mild dizziness and “near-syncope” during the summer months and developed pain in her fingers during the winter months akin to Raynaud's syndrome. After about two years of her married life in the United States, she decided to go for a neurology consultation with a POTS expert.

History of Present Illness

The patient complained of dizziness with a change in position. She denied tinnitus, hearing loss, or vertigo. The patient had nausea and dizziness. The patient felt vertigo inside of her body but not outside. She sustained three episodes of near-syncope with a sensation of darkness in front of her eyes. The patient was able to avoid syncopal episodes. She has reportedly had seven to 10 episodes of loss of consciousness (syncope) during her lifetime. She was diagnosed with POTS in India. The patient was evaluated with a tilt table test at that time. The patient had palpitations early in the disease process; however, it has resolved now. Overall, her condition improved over the years. The patient reported that she had not been taking medications prescribed for her POTS symptoms. She sought medical help, as she noticed a worsening of her symptoms during the past two months. She was unable to stand for a prolonged period of time. The patient was afraid to take care of her one-year-old child, anticipating possible episodes of syncope. She denied numbness, tingling sensations, or balance problems. She also denied any falls but did complain of weakness in both lower extremities.

Neurological Examination

A mental status examination revealed that the patient was alert and oriented to time, place, and person. Memory examination revealed normal short-term memory with good recall. Attention span and concentration were normal. General knowledge and information were appropriate. Speech articulation, fluency, and comprehension were normal. Naming and repetition were appropriate. The vocabulary was full. The cranial nerve examination revealed normal visual acuity and unrestricted visual fields. The color perception was normal. The pupils were rounded, equal, and reactive to light. There was no restriction of eye movements. Accommodation and conversion synkinesis were normal. There was no evidence of nystagmus. Muscles of mastication, facial muscles, sternocleidomastoid, trapezius, and tongue muscles were graded as 5/5. Facial sensation to temperature and pinprick was appropriate bilaterally. Hearing was normal bilaterally. The uvula was in the midline. Corneal, blink, jaw jerk, and gag reflexes were present bilaterally. The sensory examination was performed with pinprick and light touch and was found equal bilaterally. Vibration and proprioception were equal bilaterally. Neurological examination revealed diminished vibratory sense in both lower extremities.

Motor and Cerebellar Functions

The muscle tone and bulk were normal. Muscle strength was graded as 5/5 in all muscle groups in the upper and lower extremities. Deep tendon reflexes were two at the biceps, triceps, knees, and ankles bilaterally. Plantar reflexes showed downgoing toes bilaterally. Rapid alternating movement examination was normal. There was no ataxia or dysmetria. The finger-to-nose test and heel-knee-shin test were normal bilaterally. Gait was normal. The Romberg sign was normal with eyes open and closed. The patient was able to perform tandem gait, tip-toe walking, and heel walking.

Electrodiagnostic and Neurophysiological Evaluation

As the patient presented with longstanding symptoms of postural orthostatic tachycardic syndrome, lower extremity paresthesia, and diminished vibratory sense in both feet, it was necessary to rule out peripheral polyneuropathy. The neurophysiological evaluation did not reveal large fibers.

Other Details

The patient’s medical history was unremarkable. Her surgical history was significant for her cesarean section a year ago. No known allergies were reported. The patient was not currently on any medications. There was no history of alcohol, tobacco, or substance use. The patient’s father had a history of myocardial infarction, and the mother had a history of kidney problems and removal of one kidney. A review of the systems revealed palpitations, lightheadedness, constipation, and heat intolerance. Physical examination revealed a cooperative, well-nourished, afebrile patient. There was no evidence of carotid bruits. Funduscopic examination revealed flat discs without evidence of hemorrhage. Her other parameters were as follows: supine blood pressure: 126/80 mmHg, heart rate: 96 bpm, and standing blood pressure: 122/86 mmHg.

Diagnostic Impression

After reviewing abundant medical records from India, the following impressions were made: (i) confirmation of the diagnosis of POTS; (ii) there was no evidence for large fiber peripheral polyneuropathy; and (iii) consideration should be given to autonomic neuropathy.

Recommendation

For further evaluation and opinion from specialists, the patient was referred to a cardiologist and a neurologist who had expertise and experience in autonomic disorders. The patient was prescribed 5 mg of midodrine at 8 am to control blood pressure. The patient was advised to record her blood pressure on a daily basis using a blood pressure monitor. In addition, she was advised to continue lower extremity exercises and increase her oral fluid and salt intake. The patient was also advised that she might benefit from pressure stockings and abdominal binders as well as from sleeping in the Trendelenburg position with her head elevated. As advised, the patient consulted a cardiologist and a neurologist, but no significant symptoms or findings other than those noted in the history of the present illness were identified.

Current status (at age 51)

Over the past 18 years in the United States, since her previous hospital visit at about age 33, the patient has been continuously taking metoprolol (50 mg/day) and maintaining well. Apart from her mild symptoms related to thermoregulation (vasodilation to warm weather and vasoconstriction similar to Raynaud's phenomenon and chilblains in her feet during cold weather), she has been functioning optimally in all activities of daily living and occupation. For example, she could drive her car and perform household chores, besides the successful completion of her United States Medical Licensing Examination (USMLE) exams and optimal occupational functioning in medical settings.

## Discussion

We report the case of a 21-year-old young woman who developed symptoms of POTS four weeks after complete recovery from acute malignant malaria that lasted for three weeks. She had palpitations, low blood pressure, dysautonomia, and syncope (Table 1 presents a list of system-wise symptoms as reported by Fedorowski [17]). She was diagnosed with POTS at the age of 25 during her first visit to a multispecialty hospital. Due to continuous treatment, she recovered well from POTS symptoms and managed to live with mild symptoms of dysautonomia until age 31. The dysautonomic symptoms re-emerged following two years of being off medication, which prompted her to seek another hospital visit and further referrals as they became less tolerable. She was back on regular medication and has continued to maintain well until now, at age 51. This case was one of the first POTS cases in India, diagnosed 25 years ago. Unlike the widely reported cases of post-viral POTS diagnosis, this is one of the rare cases of POTS due to post-malarial infection.

**Table 1:** Clinical symptoms of postural orthostatic tachycardia syndrome involving different systems as described by Fedorowski Reproduced from the Journal of Internal Medicine, Volume 285, Fedorowski A, "Postural orthostatic tachycardia syndrome: clinical presentation, aetiology and management", Pages 352-366, Copyright 2018, with permission from John Wiley and Sons [17]. ©2018 The Association for the Publication of the Journal of Internal Medicine

System	Symptoms
General or multiple systems	General deconditioning, chronic fatigue, exhaustion, heat intolerance, fever, debility, bedriddenness
Cardiovascular system	Main: orthostatic intolerance, orthostatic tachycardia, palpitations, dizziness, lightheadedness, presyncope, exercise intolerance; Other frequent symptoms: dyspnea, chest pain/discomfort, acrocyanosis, Raynaud's phenomenon, venous pooling, limb edema
Nervous system	Headache/migraine, mental clouding (‘brain fog’), cognitive impairment, concentration problems, anxiety, tremulousness, light and sound sensitivity, blurred/tunnel vision, neuropathic pain (regional), sleeping disorders, involuntary movements
Musculoskeletal system	Muscle fatigue, weakness, muscle pain
Gastrointestinal system	Nausea, dysmotility, gastroparesis, constipation, diarrhea, abdominal pain, weight loss
Respiratory system	Hyperventilation, bronchial asthma, shortness of breath
Urogenital system	Bladder dysfunction, nycturia, polyuria
Skin	Petechiae, rashes, erythema, telangiectasias, abnormal sudomotor regulation, diaphoresis, pallor, flushing

Interestingly, similar to post-viral POTS, malaria-related POTS may cause neurological damage. Severe orthostatic hypotension associated with tachycardia and insufficient peripheral vasoconstriction, as often manifested in POTS, has been widely reported in acute malignant malaria caused by *Plasmodium falciparum* [18], although it is very rarely described in uncomplicated malaria due to *Plasmodium vivax* [19]. To our knowledge, while malaria-related POTS has not been reported so far, post-malaria neurological syndrome with acute disseminated encephalomyelitis (ADEM) has been reported in a case report of a 30-year-old woman [16], who had a multifocal, monophasic, demyelinating disease akin to a post-viral infection. It is also possible that antimalarial drugs such as quinine might cause neural damage. A rat study reported that mefloquine, a quinoline-methanol derivative of quinine, caused profound alterations in the autonomic and respiratory control systems [20]. A human cell culture study revealed that the antimalarial drugs quinine, chloroquine, and mefloquine act as antagonists at 5-HT3 receptors [21]. Therefore, it is highly likely that acute neural damage followed by chronic dysfunction of the autonomic nervous system following infection caused by Plasmodium falciparum and anti-malarial treatment is a possibility in our patient, although there were no MRI and other imaging studies done to support this claim.

In one of the largest studies on 4835 participants with POTS, Shaw et al. [5] reported that most of the patients with POTS were identified as female (80%-94%), ages between 12-50 years with a median age of 17 years, and 47% developed first symptoms after the age of 18. The major causes reported were infection (41%), surgery (12%), pregnancy (9%), vaccination (6%), accident (6%), puberty (5%), post-concussion (4%), and emotional stress or trauma (3%). The study also reported that patients waited a median of two years to get a diagnosis with a mean of 4.9 ± 7.1 years, reflecting extraordinarily long waits. Further, 21% (n = 627) of patients reported having seen more than 10 doctors until they were diagnosed with POTS. In our patient, the symptoms started at age 21, following acute malignant malaria, and it took over four years to get the diagnosis of POTS, as it is understandable that 75% of patients reported being misdiagnosed before receiving the diagnosis of POTS even in recent times [5]. Although orthostatic tachycardia, the cardinal feature of POTS, was first described in 1940 [22], it was systematically described as a diagnostic category by Schondorf and Low in 1993 [23], just two years before our patient received the diagnosis. Postural orthostatic tachycardia syndrome is a heterogeneous dysautonomic disorder with various subtypes [24], which is often underdiagnosed because symptoms mimic many related disorders, including vasovagal syndrome [4]. Interestingly, to our knowledge, there are no studies or case reports available for the post-malarial POTS, as described in our case study.

The three most common symptoms of POTs were lightheadedness, tachycardia, and presyncope, a symptom triad that is present in 91% of the patients [5]. Our patient had all three symptoms, along with headache, nausea, vomiting, blurry vision, dizziness, and syncope, until she was diagnosed and treated with medication. Shaw et al. [5] reported only 16% (N = 610) of POTS patients had Raynaud's phenomenon, which was also present in our patient and persisted for about 30 years even after being on continuous medication with metoprolol, a beta-blocker, as these medications have no role in controlling excessive vasoconstriction. However, nifedipine was prescribed to counteract Raynaud’s phenomenon as needed. Although there are no FDA-approved drugs available for treating patients with POTS, individualized treatment strategies are typically based on different subtypes of POTS, such as hypovolemic, neuropathic, and hyperadrenergic subtypes. A systematic review [3] reported that the pharmacological approach for treating POTS is usually specific to its symptom clusters: (i) midodrine for reducing orthostatic symptoms, (ii) bisoprolol and fludrocortisone for orthostatic intolerance, and (iii) beta-blockers such as metoprolol for palpitations and tremors (Table 2 presents a list of the most widely used pharmacological treatment for POTS as reported by Fedorowski [17]). These pharmacological treatments help improve blood flow and reduce symptoms such as lightheadedness, dizziness, and fatigue, which are common in patients with POTS, by regulating the autonomic nervous system, which controls involuntary functions of the body, including heart rate, blood pressure, and digestion [3]. Non-pharmacological and lifestyle changes, such as salt intake, exercise training, food regulation, compression stockings/garments, and avoiding prolonged standing, exertion, and extreme temperatures, are also important in controlling the symptoms of POTS [17]. Interestingly, 52% of the POTS patients attributed symptom improvements to nonpharmacological treatments [5]. Our patient, who is a medical doctor, properly followed both pharmacological and non-pharmacological treatments with the guidance of her clinical team, and therefore, she has been able to function optimally in various life settings over the past 30 years. Interestingly, as in our patient, a relatively recent case report describes the delay and difficulty in receiving the POTS diagnosis in a male medical student, who was later successfully treated with proper medications [25].

**Table 2:** The most widely used pharmacological treatment for POTS (as reported by Fedorowski) TID: three times a day; BID: two times a day; BP: blood pressure; POTS: postural orthostatic tachycardia syndrome Reproduced from the Journal of Internal Medicine, Volume 285, Fedorowski A, "Postural orthostatic tachycardia syndrome: clinical presentation, aetiology and management", Pages 352-366, Copyright 2018, with permission from John Wiley and Sons [17]. ©2018 The Association for the Publication of the Journal of Internal Medicine

Drug	Comments
Heart rate-controlling agents	
Beta-blockers (propranolol, 10-40 mg TID; bisoprolol, 2.5-5 mg BID; metoprolol, 25-100 mg daily; atenolol, 12.5-50 mg daily)	Beta-blockers are especially recommended in the ‘hyperadrenergic’ subtype associated with sinus tachycardia >120 bpm on standing. Beta-blockers may aggravate orthostatic intolerance in low-BP phenotypes, asthma, and paroxysmal chest pain.
Ivabradine (2.5-7.5 mg BID)	This drug is effective in low-BP phenotypes or when beta-blockers are not well tolerated. It is usually seen as an alternative to beta-blockers.
Verapamil (40-80 mg BID/TID)	This calcium channel blocker with a negative chronotropic effect can be tested in the ‘hyperadrenergic’ type associated with higher BP, migraine, and chest pain.
Vasoactive and volume-expanding agents	
Clonidine (0.2-0.6 mg BID)	Centrally acting α2-adrenoreceptor agonist with overall sympatholytic effect. It is generally recommended for the ‘hyperadrenergic’ subtype and hypertensive tendency on standing.
Midodrine (2.5-10 mg TID)	Direct α1-adrenoreceptor agonist. One of the few pharmacological agents positively tested in placebo-controlled studies for orthostatic hypotension. It may be effective in the ‘hypovolemic’ subtype and low-BP phenotype with pronounced orthostatic intolerance.
Droxidopa (100-600 mg TID)	Peroral norepinephrine precursor. The drug has been empirically used off-label in severe POTS.
Pyridostigmine (30-60 mg BID/TID)	Acetylcholinesterase inhibitor. It might be considered in the POTS phenotype associated with suspected autonomic neuropathy, gastrointestinal dysfunction, and non-specific muscle weakness. The effect on BP is small.
Fludrocortisone (0.1-0.2 mg daily)	Mineralocorticoid. Volume expander. Increases sodium reabsorption and enhances the sensitivity of α-adrenoreceptors. The drug may worsen supine hypertension and hypokalemia. It is recommended in the ‘hypovolemic’ subtype and low-BP phenotype.
Ephedrine and pseudoephedrine (25/30-50/60 mg TID)	Direct and indirect α1-adrenoreceptor agonist. Efficacy is controversial.
Desmopressin (0.1-0.4 mg BID)	Vasopressin analog. Volume expander. Increases water reabsorption and reduces nycturia. Sparse evidence exists. Efficacy is uncertain.
In-hospital acute 1-2 L physiological saline infusion (during consecutive 3-5 days)	In acute decompensated POTS, this method should be considered to alleviate the short-term symptoms.

Although POTS has been a highly recognized diagnostic category, especially since the COVID-19 pandemic, due to its high prevalence following SARS-CoV-2 infection [15], it is also important to study malaria-related POTS, especially in tropical and poor countries where malaria infections are common. Although there were no obvious neurological manifestations observed due to malignant malaria or malarial treatment in our patient, both animal and human studies have demonstrated neuronal damage following severe malarial illness. Our case study highlights the fact that post-malarial POTS seems to be a neglected area of research, as there is a lack of data and a paucity of literature on this topic. While the diagnosis and management of POTS, irrespective of causal factors, are practically the same in current practice, we propose that the pathophysiology and manifestations of these symptoms may vary subtly due to specific etiological mechanisms. We also recommend that strategies aimed at both preventing and treating POTS symptoms should include both pharmacological and non-pharmacological measures, as both domains have been shown to reduce symptoms and improve functioning, as our case report suggests.

## Conclusions

We present a case study of a 21-year-old medical student who presented with prominent symptoms of dysautonomia after suffering from an acute malarial infection. Following the confirmation of POTS by a tilt table test done after four years, regular medication along with lifestyle modifications led to a fast recovery from major symptoms and better functioning and quality of life. To our knowledge, this is the first case report of a non-viral and post-malarial onset of POTS that occurred long before the COVID-19 pandemic. It is recommended that many more studies be conducted to understand the manifestations and pathophysiology of the syndrome based on its etiological origin. Awareness programs targeted to the public as well as training programs designed for medical professionals to diagnose and treat POTS can mitigate the challenges in arriving at an early diagnosis and effective management strategies for this complex, multi-system disorder.
